# From Values to Action: An Integrative Explanatory Framework for Insect Conservation Intentions and Behavior

**DOI:** 10.3390/insects16121274

**Published:** 2025-12-15

**Authors:** Geanina Magdalena Sitar, Ivana Ostřanská Spitzer, Lukas Spitzer, Claudia Marian, Iulia Francesca Pop, Cristian Sitar, Alina Simona Rusu

**Affiliations:** 1 Doctoral School “Education, Reflection, Development”, Faculty of Psychology and Educational Sciences, Babes-Bolyai University, 7 Sindicatelor Street, 400029 Cluj-Napoca, Romania; geanina.iacob@ubbcluj.ro; 2 Museum of the Moravian Wallachia Region, Horní náměstí 2, 755 01 Vsetín, Czech Republic; ostranska@muzeumvalassko.cz (I.O.S.); spitzer.lukas@gmail.com (L.S.); 3 Biology Centre of the Czech Academy of Sciences, Institute of Entomology, Branišovská 31, 370 05 České Budějovice, Czech Republic; 4 Specialized Department with a Psychopedagogical Profile, Technical University of Cluj Napoca—North University Center of Baia Mare, Victoriei Street, 76, 430113 Baia Mare, Romania; claudia.marian@dspp.utcn.ro; 5 Department of Fundamental Sciences, Faculty of Animal Science and Biotechnologies, University of Agricultural Sciences and Veterinary Medicine, 3–5 Calea Mănăştur, 400372 Cluj-Napoca, Romania; iulia-francesca.pop@usamvcluj.ro; 6 Human-Animal Interaction Research Lab, Faculty of Animal Science and Biotechnologies, University of Agricultural Sciences and Veterinary Medicine, 3–5 Calea Mănăştur, 400372 Cluj-Napoca, Romania; 7 Zoological Museum, Babes-Bolyai University, 5–7 Clinicilor, 400006 Cluj-Napoca, Romania; 8 Department of Cluj, Emil Racovita Institute of Speleology, Clinicilor 5, 400006 Cluj-Napoca, Romania

**Keywords:** human–insect relationships, insect conservation behavior, attitudes towards insects, Theory of Planned Behavior, Value–Belief–Norm Theory

## Abstract

Insects constitute a vital component of terrestrial ecosystems, yet their ongoing global decline underscores the urgency of identifying the factors that facilitate or hinder public engagement in their conservation. This study identifies the key psychological drivers of insect-related conservation behavior within a Romanian context, an understudied geographical and sociocultural setting. Using data collected from 346 adult respondents via an online questionnaire, the predictive performance of the Value–Belief–Norm (VBN) theory, the Theory of Planned Behavior (TPB), and an integrated VBN–TPB framework was examined through Partial Least Squares Structural Equation Modeling (PLS-SEM). The VBN model exhibited superior explanatory power relative to TPB, with biospheric values, ecological worldviews, and personal moral norms emerging as the most influential determinants of behavioral intention and self-reported action. Although participants demonstrated moderate levels of general entomological knowledge, awareness of specific insect-friendly practices was notably limited and frequently characterized by misconceptions. Perceived barriers, particularly informational deficits, time constraints, and financial considerations, exerted significant inhibitory effects on conservation engagement. The findings indicate that effective interventions must extend beyond knowledge transmission, incorporating strategies that activate moral norms, strengthen affective and identity-based motivations, and reduce structural barriers to action.

## Simple Summary

Insects are disappearing at an alarming rate around the world, even though they are essential for pollination, soil health, food webs, and many other natural processes that humans depend on. Protecting insects requires not only scientific solutions, but also public understanding and willingness to act. This study investigated what people in Romania know about insects, how they feel about them, and what motivates or prevents them from taking action to protect insect species. We surveyed 346 adults using an online questionnaire and examined how different beliefs, values, and personal motivations influence conservation behavior. The results show that people are more likely to help insects when they feel a sense of moral responsibility, believe insects are important for nature, and see pro-environmental action as part of who they are. However, many participants did not know which everyday actions actually support insect conservation, and common barriers, such as lack of information, time, or financial resources, reduced motivation to act. These findings suggest that simply providing facts is not enough. Conservation programs must also address values, emotions, and practical obstacles. Making insect protection easier, clearer, and personally meaningful can strengthen public participation and support long-term biodiversity protection.

## 1. Introduction

The relationship between humans and nature has evolved throughout history. In early societies, people regarded themselves as part of the natural world and depended directly on its resources. With technological and economic development, this relationship became primarily utilitarian, and nature has come to be viewed as a source of materials and energy [1,2]. As a result, the interdependence of ecosystems and the need for their protection were often overlooked.

Current evidence indicates that the planet faces several environmental crises, most of which are caused by human activity [3,4,5]. Overexploitation, deforestation, mining, pollution, and habitat fragmentation have accelerated ecosystem degradation [6,7]. These processes affect not only human well-being, but also the survival of numerous plant and animal species, emphasizing the need for sustainable management and environmental education [8,9,10]. Although international policies aim to conserve biodiversity and restore ecosystems [11,12,13], their effectiveness depends on public understanding and participation. Social research shows that individual actions, even when small in isolation, can have cumulative effects on ecological systems [14]. Promoting environmentally responsible behaviour therefore requires both structural measures and educational approaches that can influence social norms and attitudes [15,16].

Among the components of biodiversity currently under pressure, insects hold a distinctive position. They are essential to ecosystem functioning but remain undervalued in public perception. Insects represent the most diverse animal group on Earth and play key roles in processes such as pollination, decomposition, and soil formation [17,18]. Nevertheless, global assessments report substantial declines in insect populations in recent decades [19,20]. Despite their ecological significance, insects and other invertebrates receive limited attention in conservation policies and research, accounting for around 10% of funding although they represent about 80% of known animal species [21,22]. Public perception contributes to this imbalance [23,24]. While large vertebrates such as mammals and birds are generally appreciated, insects are often linked to fear or discomfort [25,26,27]. Such perceptions can restrict public engagement and reduce support for conservation initiatives [28]. As a result, insects, with some exceptions (e.g., butterflies and honeybees), and other small taxa receive less attention and fewer conservation resources [21,29]. Familiarity, education, and cultural representations influence how people react to insects [30,31]. Because most individuals encounter only a limited number of species, their attitudes are strongly influenced by early experiences and educational exposure [32]. The reduction in direct interaction with nature, a process referred to as the “extinction of experience” [33,34], has further limited opportunities for such contact, especially among younger generations [35,36]. Environmental education can mitigate this tendency by offering direct experiences with nature. When education includes practical or experiential components, it can foster understanding and empathy toward living organisms, including insects [37,38,39]. Evidence indicates that values and beliefs acquired through education influence environmental attitudes and behaviours later in life [40,41].

In biodiversity communication, the use of “flagship” species is a common strategy. Butterflies, bees, and ants tend to elicit positive associations, whereas other taxa such as flies or cockroaches are often viewed negatively. Schlegel et al. [42] showed that other local invertebrates can also serve as ambassadors for conservation if they are ecologically relevant and easily recognizable.

Recent research highlights that effective environmental communication depends not only on the accuracy of the information conveyed, but also on the way it is framed and delivered [43,44]. In this context, storytelling has been proposed as a promising strategy for conservation education, as it can translate scientific knowledge into emotionally resonant and relatable narratives [45,46,47]. The choice of language, tone, and communicative framing can shape public understanding, receptiveness, and willingness to engage with environmental issues [44]. Consequently, outreach and educational programs may benefit from interdisciplinary collaboration among specialists in biology, ecology, the social sciences, and the arts, in order to produce messages that are both scientifically grounded and culturally accessible, thereby enhancing awareness and fostering meaningful community participation.

### 1.1. Human–Insect Relationships

Despite their ecological importance, human behaviour toward insects is still not well understood. Attitudes, beliefs, and emotional responses toward insects influence conservation outcomes, yet this topic remains less explored than other aspects of biodiversity [21,28]. Understanding these psychological and cultural dimensions is essential for developing strategies that promote sustainable human–insect interactions.

Human behaviour is a major factor shaping environmental outcomes and is influenced by values, social norms, and situational factors [15,16,48]. Pro-environmental behaviour refers to intentional actions that reduce environmental impact [49,50]. While early models proposed a direct link between knowledge, attitudes, and behaviour [51,52], subsequent research emphasized the moderating role of cognitive and social variables [52]. Within environmental psychology, the Theory of Planned Behaviour (TPB) [48] and the Value–Belief–Norm (VBN) framework [53] explain how attitudes, values, beliefs, and perceived behavioural control shape environmental decisions.

The TPB, a psycho-social framework rooted in psychology and sociology, is grounded in principles of rational choice and self-interest, proposing that individuals engage in deliberate reasoning when deciding whether to perform a particular behavior [48]. As an extension of the Theory of Reasoned Action [15], TPB was developed to address the limitations of the original model in explaining behaviors that are not entirely under volitional control. It posits that behavioral intention, the most immediate determinant of action, is shaped by three key components: *attitude*, or the individual’s favorable or unfavorable evaluation of the behavior; *subjective norm*, representing perceived social pressure to perform or abstain from the behavior; and *perceived behavioral control*, referring to one’s perception of the ease or difficulty associated with the behavior. Empirical studies have consistently shown that individuals with more positive attitudes, stronger perceived social support, and greater confidence in their ability to act are more likely to form intentions that lead to actual behavioral engagement [48,54]. The TPB framework has been widely applied in environmental research to explain behaviors such as water conservation behavior [55,56], recycling [57,58,59], forestry management practices [60], energy-saving behavior in workplaces [61,62,63,64] and adoption of low-carbon technologies by homeowners [65].

The VBN model, developed by [16,53], synthesizes three theoretical foundations: the Universal Theory of Human Values [66], the Norm Activation Model [67,68], and the New Environmental Paradigm (NEP) [69], which represents a general ecological worldview. The model posits a linear causal chain in which basic value orientations (*biospheric*, *altruistic*, *or egoistic*) shape individuals’ endorsement of the NEP [16,53,70], which then influences their *awareness of the consequences* of human action, followed by the *ascription of responsibility*, the activation of *personal norms*, and ultimately the performance of pro-environmental behavior. In this sequence, NEP functions as the belief-level mediator that links value priorities to more specific environmental problem perceptions, thereby enabling the transition from abstract values to concrete moral obligation [16,53,69]. Once activated, personal norms, defined as an internalized sense of moral duty, serve as the most immediate predictor of pro-environmental actions. Although the VBN model has been extensively employed in diverse domains, including environmental conservation [71], sustainable tourism [72], and willingness to pay for the conservation [73], empirical evidence indicates that it explains moral or normative motivations more robustly than overt behavioral outcomes [72,73,74].

Considering the conceptual complementarities between the two models, TPB emphasizing rational intention formation and VBN highlighting value-driven moral obligation, scholars have increasingly argued for their integrative application in the study of pro-environmental action. In this line of research, integrative frameworks have been proposed to incorporate both rational–cognitive predictors and value-based normative drivers, thereby offering a more comprehensive explanation of behavioral variance [75,76,77].

Previous research has highlighted the central role of public awareness, knowledge, and attitudes in shaping conservation-oriented behaviors, yet studies focusing specifically on insects remain scarce [21,23,28]. To date, only one empirical study [78] has examined human–insect relationships through the lens of the TPB, and no prior research has employed an integrated framework combining the TPB and VBN theories in this context. In response to this gap, the present study proposes a comprehensive integrative model designed to investigate the determinants of responsible behavior toward insects. The model merges the key constructs of the TPB and VBN theories and extends them by incorporating five additional variables considered relevant to the human–insect relationship: *knowledge level*, *naturalist identity*, *nature connectedness*, *perceived barriers*, and *perceived opportunities*.

### 1.2. Research Aims

The primary aim of this study was to determine which theoretical framework best explains human behavior toward insects in Romania by comparing three well-established models: the VBN model, the TPB model, and an integrated VBN–TPB model. Additionally, this study aimed to report the knowledge levels, attitudes toward insects, and perceived barriers within the sample. Through this approach, the study aims to advance theoretical understanding of the psychological and contextual factors that foster or inhibit pro-insect behavior.

Accordingly, the specific objectives of the study were:

1. To analyze the attitudes toward insects in relation to individual and contextual factors.
2. To examine the level of knowledge about insects and to identify the main factors influencing it.
3. To investigate perceived barriers to the adoption of responsible insect-related behaviors according to key demographic variables.
4. To identify the theoretical model, among the TPB, VBN, and integrated VBN–TPB frameworks, that most effectively explains responsible behavior toward insects in the analyzed sample.

### 1.3. Hypotheses

**H1:**  *The combined VBN–TPB model will outperform the single-framework models (VBN-only, TPB-only) in explaining behavioral intention and behavior (*i.e.*, higher R^2^ and predictive relevance, Q^2^). Evidence shows that integrating TPB with moral or normative variables from VBN improves predictive power [75,76].*

Beyond this baseline comparison, the present study also adopted an exploratory approach by including additional psychological and contextual variables shown in prior research to be relevant for pro-environmental behaviors. These include the following variables: knowledge [79,80,81], nature connectedness [82,83], naturalist or environmental identity [84,85], and situational factors such as perceived barriers and perceived opportunities [86,87].

Insect-focused studies further underscore the importance of these constructs: knowledge predicts more favorable attitudes toward pollinators and conservation [88] while misconceptions sustain fear and negative perceptions [31,89].

**H2.**  *Knowledge about insects is expected to positively influence psychological antecedents of pro-environmental action [90,91]:*

**H2a.**  *Knowledge positively predicts environmental attitudes.*

**H2b.**  *Knowledge positively predicts perceived behavioral control, thereby indirectly supporting pro-environmental behavior.*

**H3.**  *Nature connectedness is hypothesized to enhance both biospheric orientations and evaluative responses toward the environment [92,93]:*

**H3a.**  *Nature connectedness positively predicts biospheric values, as greater connectedness has been shown to strengthen biospheric concern.*

**H3b.**  *Nature connectedness positively predicts environmental attitudes, reflecting a tendency toward stronger pro-environmental orientations.*

**H4.**  *Naturalist identity is expected to reinforce value- and attitude-based predictors [84,85]:*

**H4a.**  *Naturalist identity positively predicts biospheric values, consistent with the role of environmental identity in conservation motives.*

**H4b.**  *Naturalist identity positively predicts environmental attitudes, in line with findings that ecological identity fosters stronger environmental concern.*

**H5.**  *Situational factors are hypothesized to constrain or facilitate behavioral engagement [94,95]:*

**H5a.**  *Perceived barriers negatively predict behavioral intention.*

**H5b.**  *Perceived barriers negatively predict behavior, reflecting their role as obstacles to action.*

**H5c.**  *Perceived opportunities positively predict behavioral intention.*

**H5d.**  *Perceived opportunities positively predict behavior, as supportive contexts enable the translation of values and norms into action.*

## 2. Materials and Methods

### 2.1. Questionnaire

Data were collected through a structured questionnaire specifically developed for this study, adapted from instruments previously used by [76], who operationalized constructs from the TPB and the VBN theory to explain pro-environmental behavior, and by [78], who designed a TPB-based instrument focused on pollinator conservation in the United Kingdom. The present instrument incorporated the main components of the TPB: attitudes (AT) (10 items), subjective norms (SN) (3 items), perceived behavioral control (PBC) (6 items), behavioral intention (INT) (6 items), and behavior (BEH) (24 items), and the VBN theory: biospheric values (BV) (3 items), ecological beliefs (NEP) (5 items), awareness of consequences (AC) (6 items), ascription of responsibility (AR) (3 items), and moral norms (MN) (3 items), alongside measures of knowledge about insects (KNOW) (11 items), naturalist identity (IDn) (2 items), nature connectedness (CN) (4 items), perceived barriers (BAR) (11 items) and perceived opportunities (OPP) (7 items) (Document S1).

The questionnaire was first developed in English, then translated and culturally adapted into Romanian language following a translation–back translation procedure. Its content validity was verified through expert review involving specialists in biology, ecology, educational sciences, and psychology. Examples of items are presented in Table 1, while the full version of the questionnaire is available in the Supplementary Materials (Document S1).

### 2.2. Sampling

A questionnaire survey method was employed to collect data and test the research hypotheses. Data were gathered through an online structured questionnaire administered to participants residing in Romania. The questionnaire was distributed electronically via Google Forms between August 2024 and January 2025, and participants were recruited through informal dissemination channels using a snowball sampling technique. Participation in the study was entirely voluntary, with confidentiality and anonymity explicitly ensured through an informed consent statement presented at the beginning of the survey. Although this non-probabilistic sampling approach does not permit statistical generalization, it was deemed suitable for the exploratory objectives of the present research.

### 2.3. Participants

The final sample consisted of 346 respondents. Most participants identified as female (71.4%), followed by male (25.4%), while 2% identified as another gender or preferred not to disclose. The mean age was 30.9 years (SD = 12.7), with the largest age group being 18–24 years (*n* = 185; 53.5%), followed by those aged 35–44 years (*n* = 60; 17.3%). The 25–34 and 45–54 age groups were equally represented *(n* = 40; 11.6% each).

In terms of occupational status, nearly half were students (49.7%), followed by employed individuals (41.3%). Additional categories included unemployed individuals (*n* = 11), retirees (*n* = 4), and others in alternative occupational situations (*n* = 16).

Most respondents reported living in urban areas (69.1%), while 30.1% were from rural environments. Regarding educational level, the majority of respondents indicated that their highest level of education completed was high school (*n* = 136; 39.3%), followed by a bachelor’s degree (*n* = 98; 28.3%) and a master’s degree (*n* = 64; 18.5%). More advanced levels of education, such as doctoral (*n* = 15) and postdoctoral studies (*n* = 11), were reported less frequently. A small number of participants reported post-secondary or technical education (*n* = 6) or vocational qualifications (*n* = 5). Additionally, eleven respondents (3.2%) chose not to disclose their educational background.

Income distribution was heterogeneous: 27.1% of respondents reported earnings below 300 EUR/month, while 18.4% and 11.5% fell within the 300–500 EUR and 500–700 EUR ranges, respectively. Another 19.8% reported a monthly income between 700–1000 EUR, and 13.2% earned between 1000–1500 EUR. Only a small proportion of participants (10%) reported incomes above 1500 EUR/month, including 4.2% earning 1500–2000 EUR, 2.1% earning 2000–3000 EUR, and 3.8% earning more than 3000 EUR/month.

Regarding respondents’ access to contexts that enable direct engagement in nature-related or land-based activities, 60.4% indicated that they had at least one such opportunity, whereas 39.6% reported none. The most common form of engagement was the ownership or management of a private garden (55.5%), followed by the administration of agricultural land (18.2%) and the ownership or lease of land under personal management (16.5%). Only a small proportion of respondents reported more specialized forms of involvement, such as the management of organized green spaces (2.0%), beekeeping (1.4%), or operating a business with an ecologically managed outdoor area (1.2%).

### 2.4. Data Analysis

#### 2.4.1. Initial Data Processing

The preliminary data handling and the descriptive statistical analyses were conducted in Python 3.8 [96], together with the Pandas [97], NumPy [98], and SciPy [99] libraries. Visual representations of the distributions were created using the Matplotlib library 3.7 [100]. Likert-type items designed to assess perceptions were coded on a 7-point scale, ranging from 1 (“strongly disagree”) to 7 (“strongly agree”). Composite Likert scales were computed by summing the items corresponding to each construct (see Table 1). For each variable, including the theoretical components of TPB and VBN theories, scores from all relevant items were aggregated to yield a single composite value.

The categorization of attitudes and knowledge followed the framework proposed by [78], ensuring conceptual consistency with their approach. In their study, the authors distinguished between two types of attitudes: attitude toward pollinators and attitude toward pollinator conservation behavior. In the present study, this categorization was adapted to the broader context of insects, resulting in the constructs of attitudes toward insects (ATi) and attitudes toward insect-related behavior (ATb). Knowledge levels were assessed using two sets of true/false items. The first set (six items) measured knowledge about insects, while the second set (five items) focused on knowledge of behaviors beneficial to insect populations. Correct answers were coded as 1, and incorrect answers as 0, yielding a total possible score of 11. For descriptive purposes, knowledge scores were categorized into three levels: low (0–3 points), moderate (4–7 points), and high (8–11 points), reflecting increasing degrees of factual understanding.

Actual pro-environmental behaviors were measured using 24 behavioral statements (e.g., “I plant species beneficial to insects”, “I avoid using pesticides”). Participants indicated which behaviors they currently perform, and an overall behavioral index was computed by summing the number of reported pro-conservation actions. This approach was adopted because, in the case of insect-related behaviors, no single representative behavior could be identified; instead, a range of concrete actions was used to capture behavioral engagement in a multidimensional and ecologically valid way.

To explore relationships among variables, attitude scores were analyzed using Pearson correlations and independent-samples *t*-tests. Knowledge scores were compared across demographic groups using one-way ANOVAs with post hoc Tukey HSD tests, while correlations were applied to assess relationships with other psychological constructs. Perceived barriers were initially analyzed descriptively, followed by t-tests and one-way ANOVAs across demographic categories. In addition, Pearson correlations and simple linear regressions were employed to examine the relationship between perceived barriers and pro-conservation behaviors.

#### 2.4.2. Model Analysis

The proposed relationships were tested using Partial Least Squares Structural Equation Modeling (PLS-SEM), an approach well-suited for exploratory research and complex models with multiple latent variables. Analyses were performed using SmartPLS software (version 4.1.1.5) [101].

The evaluation followed the standard two-step procedure recommended in the PLS-SEM literature [102]. In the first step, the measurement model was assessed. Indicator reliability was considered adequate when outer loadings exceeded 0.70; loadings between 0.40 and 0.70 were retained only if the construct still met the required levels of composite reliability (CR) and Average Variance Extracted (AVE). Convergent validity was confirmed when the AVE reached the recommended minimum of 0.50. Internal consistency was examined using both Cronbach’s alpha and CR, with values above 0.70 indicating satisfactory reliability. Discriminant validity was tested using the Fornell–Larcker criterion and the Heterotrait–Monotrait ratio (HTMT), where HTMT values below 0.85 (strict criterion) or 0.90 (liberal criterion) were deemed acceptable [103].

Perceived Barriers was modelled as formative, given that its indicators represent distinct obstacles that jointly define the overall level of perceived barriers. Outer weights were examined to assess the relative contribution of each item (Table S1). Opportunities were measured as the total number of facilitating situations endorsed by each respondent and therefore treated as a single-indicator construct in the model.

Second, the structural model was examined by evaluating path coefficients and their significance using bootstrapping with 5000 subsamples, as well as the model’s explanatory power (R^2^) and predictive relevance (Q^2^). R^2^ values of 0.25, 0.50, and 0.75 indicate weak, moderate, and substantial explanatory power [102]. Predictive relevance was assessed via the blindfolding procedure (omission distance = 7) to compute Stone–Geisser’s Q^2^; values > 0 indicate predictive relevance, with 0.02, 0.15, and 0.35 reflecting small, medium, and large predictive accuracy [104,105]. For the additional exploratory constructs, multiple paths were initially tested, but only those with statistically significant coefficients and incremental explanatory contribution were retained in the final model.

#### 2.4.3. Model Specification

Given that the two theoretical frameworks identified in the literature, TPB and VBN theories, are often applied either independently or jointly to provide a more robust explanation of pro-environmental behavior, we specified three baseline models for evaluation: (1) a VBN-only model, (2) a TPB-only model, and (3) a combined model integrating constructs from both frameworks.

The initial step of the analysis was to compare these baseline models in order to identify which theoretical structure best explained insect-related behaviors in the Romanian context. The decision was based on both statistical significance of the estimated paths and the explanatory power of the model (R^2^ values).

After selecting the best-fitting baseline model, additional constructs (naturalist identity, Nature connectedness, knowledge, perceived barriers, and opportunities) were sequentially introduced. Nonsignificant paths were systematically eliminated, while retaining only those relationships that improved the explanatory power of the model. This iterative process is known in the SEM and PLS-SEM literature as model trimming [102,106,107]. The final model is thus characterized by the highest R^2^ values and a parsimonious structure of statistically significant paths.

## 3. Results

### 3.1. Descriptive Analyses of Attitudes, Knowledge, and Perceived Barriers

#### 3.1.1. Attitudes Toward Insects

Given the limited number of responses in the “other” gender categories, the gender-based analysis was restricted to male and female participants. An independent-samples *t*-test revealed a statistically significant gender difference in attitudes toward insects, with men reporting more favorable attitudes (M = 4.99) than women (M = 4.60) (t(344) = –2.44, *p* = 0.016). Furthermore, a one-way ANOVA indicated significant differences across age groups, F(5, 330) = 11.96, *p* < 0.001, showing that younger participants (18–24 years) expressed less favorable attitudes compared to older respondents. Participants’ attitudes toward insects did not vary significantly according to their place of residence (*p* = 0.693), suggesting that urban–rural context did not influence attitudinal responses. These findings suggest that attitudes toward insects are influenced not only by cognitive and affective antecedents but also by demographic characteristics, underscoring the multifaceted nature of human–insect relationships.

#### 3.1.2. Knowledge

**Figure 1:**
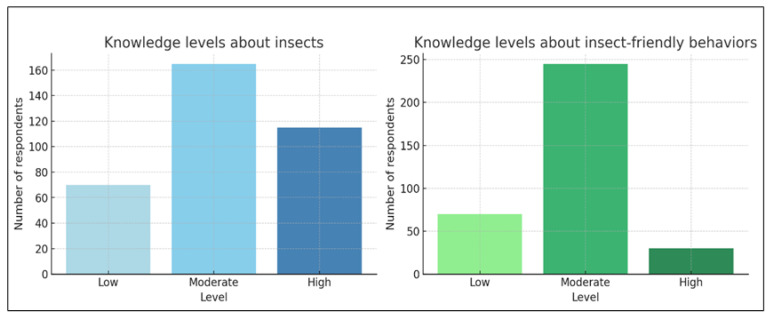
Levels of knowledge about insects and insect-friendly behaviors. Respondents showed higher levels of general insect knowledge than knowledge of insect-friendly behaviors, with the latter concentrated predominantly in the moderate range.

Most participants demonstrated a moderate level of knowledge about insects (48%), followed by high (32%) and low (20%) levels. In contrast, knowledge of insect-friendly behaviors was lower, with 71% scoring moderate, only 8% high, and 21% low (Figure 1) indicating that understanding of concrete conservation practices remains limited despite relatively adequate general knowledge.

**Figure 2:**
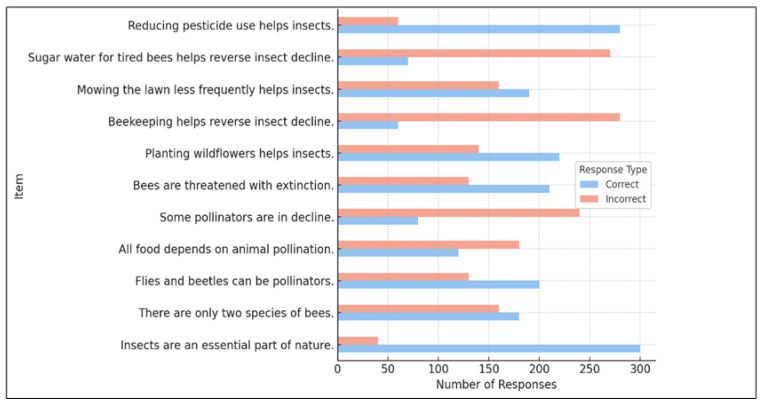
Distribution of correct and incorrect responses for the set of insect-related knowledge items, indicating heterogeneity in respondents’ conceptual understanding of insect ecology and conservation.

No statistically significant gender differences were observed in knowledge scores. However, in regard to the age of the participants, younger participants (18–24 years) demonstrated significantly lower knowledge levels compared to older groups. Although professional domain showed an overall effect, post hoc comparisons did not yield robust pairwise differences, suggesting that knowledge about insects is influenced more by general education and personal exposure than by occupational specialization. Item-level analyses further indicated notable misconceptions among respondents, such as the belief that beekeeping can reverse insect decline or that only two bee species exist (Figure 2).

**Figure 3:**
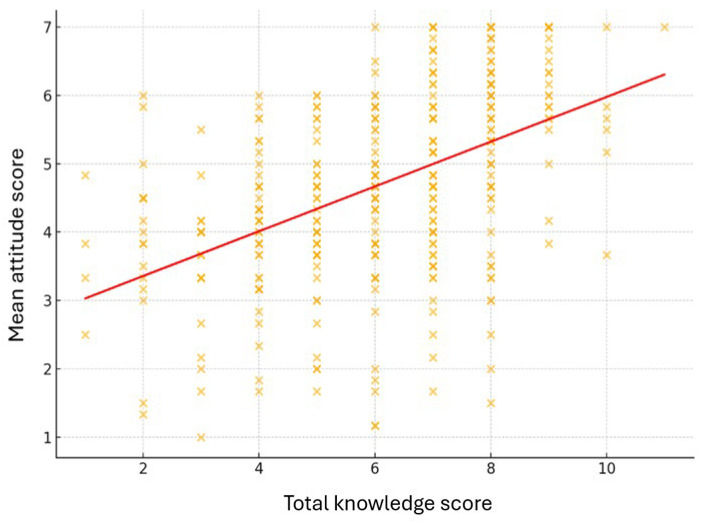
Relationship between total knowledge score and mean attitude score. Scatter plot illustrates the positive correlation between participants’ knowledge about insects and their attitudes toward insect conservation (*r* = 0.48, *p* < 0.001).

The Pearson correlation analysis revealed a moderate positive association between the variables knowledge about insects and attitudes toward their protection, r(344) = 0.48, *p* < 0.001, indicating that higher knowledge levels are associated with more favorable attitudes toward insect conservation (Figure 3).

#### 3.1.3. Perceived Barriers

The most frequently reported barriers to engaging in insect conservation behaviors were lack of knowledge (M = 3.95), lack of time (M = 3.93), and limited financial resources (M = 3.83), indicating that informational barriers are at least as constraining as logistical ones (Figure S1). Overall, the perceptions of barriers were relatively uniform across demographic groups, with no significant gender- or residence-based differences.

**Figure 4:**
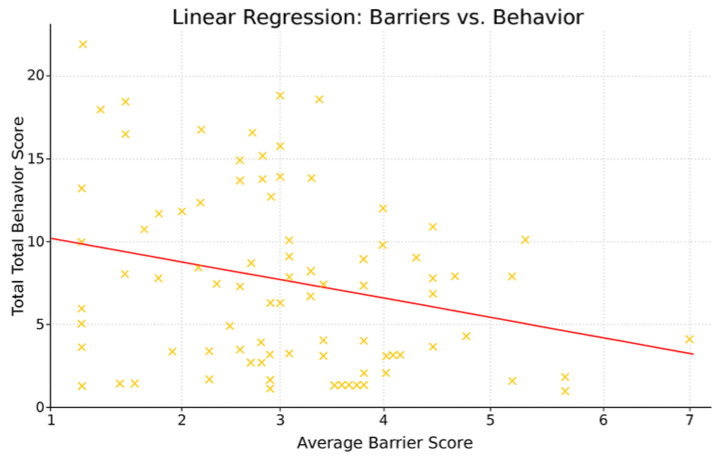
Relationship between perceived barriers and engagement in insect conservation behaviors. The scatterplot displays the negative linear association between participants’ average barrier scores and their total behavior scores, with the fitted regression line indicating that higher perceived barriers are associated with fewer reported insect-friendly behaviors. In other words, as barriers increase, participation in conservation-related actions decreases.

The results of the correlation analyses indicated a moderate negative association between the variables perceived barriers and responsible behavior toward insects (r = −0.41, *p* < 0.001) (Figure 4). A simple linear regression further confirmed that perceived barriers were a significant negative predictor of responsible insect-related behavior (β = −0.143, *p* < 0.05), explaining approximately 16.6% of the variance in self-reported conservation actions (R^2^ = 0.166).

### 3.2. Structural Model Evaluation and Hypothesis Testing

#### 3.2.1. Measurement Model Assessment

The reliability and validity of the measurement model were assessed following established PLS-SEM procedures [102]. Convergent validity was first examined through the outer loadings and average variance extracted (AVE). Most indicators exceeded the 0.70 threshold, and all AVE values were above 0.50, confirming satisfactory reliability and convergence (Table 2 and Table 3).

For the construct *ATb*, three indicators (ATb_1 = 0.848, ATb_2 = 0.725, ATb_4 = 0.826) met the loading criterion, while ATb_3 (0.399) was removed. For *ATi*, indicators ATi_1 (0.925), ATi_2 (0.830), and ATi_3 (0.933) were retained, whereas ATi_4–ATi_6 (0.517, 0.478, 0.054) were excluded to improve construct validity. For *PBC*, items 2 and 3 showed low loadings (0.208 and 0.179) and were eliminated. After these refinements, all retained indicators exceeded 0.708, ensuring that each explained more than 50% of its latent construct’s variance.

Internal consistency reliability was confirmed for all constructs except the *NEP*, which required the removal of item 5. Cronbach’s alpha and composite reliability values surpassed the 0.70 threshold, indicating satisfactory reliability (Table 1).

Elevated HTMT values were observed among *NEP*, *AC*, and *AR*, indicating conceptual overlap. However, because *BV* is theoretically distinct from belief-based constructs [14] VB was retained as a separate exogenous variable, while NEP, AC, and AR were combined as dimensions of a higher-order construct labeled *Worldview (WW)*.

To operationalize this structure, a two-stage higher-order approach was employed [108]. In the first stage, latent scores for NEP, AC, and AR were estimated; in the second, these scores were used as indicators for the Worldview construct.

In the initial specification of the TPB model, attitudes were represented by two separate constructs: ATi and ATbeh. Discriminant validity analysis showed insufficient distinction (HTMT = 0.885 > 0.85), and both constructs exhibited strong outer loadings (>0.84). This indicated that they measured the same evaluative dimension. Consequently, ATi and ATbeh were merged into a single construct, Attitudes (AT), representing general evaluative orientations toward insects and responsible behavior. This refinement improved model parsimony and statistical stability without compromising conceptual clarity.

Overall, the final measurement model retained only indicators with satisfactory reliability (outer loadings ≥ 0.708), and all constructs demonstrated acceptable levels of reliability, convergent validity, and discriminant validity (Table 3). These results confirm that the measurement model is robust and theoretically consistent, providing a reliable basis for subsequent analysis of the structural relationships between human attitudes, values, and behaviors toward insects.

#### 3.2.2. Model VBN-Only

**Figure 5:**
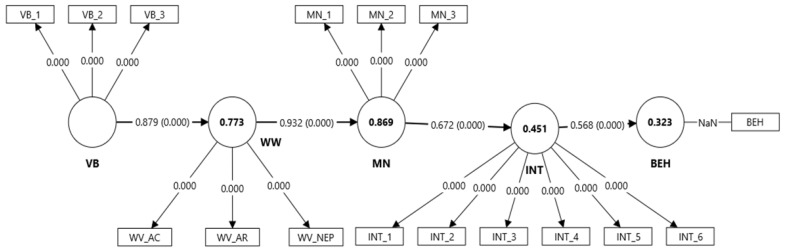
PLS-SEM structural model of the VBN framework. (BEH—behavior, INT—behavioral intention; MN—moral norms; VB—biospheric values; WW—Worldview (AC—awareness of consequences; AR—ascription of personal responsibility; NEP—New Environmental Paradigm, ecological beliefs)).

The first baseline model was specified following the VBN theoretical sequence: Biospheric values → Ecological worldview → Moral norms → Intention → Behavior. All direct paths were positive and statistically significant (VB → WW: β = 0.879, t = 50.69, *p* < 0.001; WW → MN: β = 0.932, t = 101.49, *p* < 0.001; MN → INT: β = 0.672, t = 18.38, *p* < 0.001; INT → BEH: β = 0.568, t = 14.72, *p* < 0.001) (Table 4, Figure 5). The model demonstrated strong explanatory power, accounting for 77.3% of the variance in ecological worldview (R^2^ = 0.773), 86.9% in personal norms (R^2^ = 0.869), 45.1% in behavioral intention (R^2^ = 0.451), and 32.3% in self-reported behavior (R^2^ = 0.323).

Beyond the direct relationships, specific indirect effects further confirmed the sequential mediation mechanism proposed by the VBN framework (Table 4). The indirect effect of personal norms on behavior via intention was strong and significant (MN → INT → BEH: β = 0.382, t = 11.63, *p* < 0.001), indicating that moral obligations are translated into behavior primarily through intentional processes. Similarly, biospheric values exerted substantial indirect effects on downstream constructs through a cascading chain: VB → WW → MN (β = 0.819, t = 38.18, *p* < 0.001), VB → WW → MN → INT (β = 0.550, t = 14.03, *p* < 0.001), and the full sequential mediation VB → WW → MN → INT → BEH (β = 0.313, t = 10.06, *p* < 0.001).

All endogenous constructs exhibited positive Q^2^ values, confirming the model’s predictive capability. Specifically, the model showed very high predictive relevance for ecological worldview (Q^2^ = 0.772) and personal norms (Q^2^ = 0.738), high predictive relevance for behavioral intention (Q^2^ = 0.444), and moderate predictive relevance for self-reported behavior (Q^2^ = 0.168).

#### 3.2.3. TPB-Only Model

The second baseline model was estimated according to the TPB, incorporating attitude, subjective norm, and perceived behavioral control as predictors of behavioral intention, which in turn predicted self-reported behavior.

**Figure 6:**
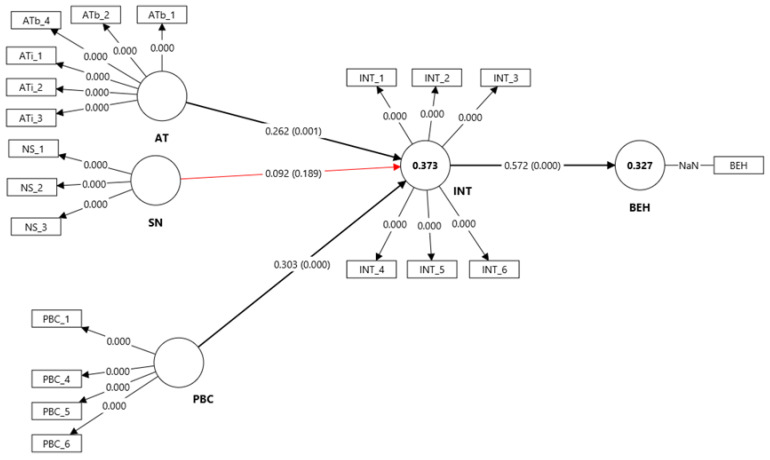
PLS-SEM structural model of the TPB framework. (BEH—behavior; INT—behavioral intention; AT—attitude; ATb—attitudes toward insect-related behavior; ATi—attitude toward insects; SN—subjective norms; PBC—perceived behavioral control).

The results indicated that attitude (β = 0.262, t = 3.25, *p* = 0.001) and perceived behavioral control (β = 0.303, t = 4.16, *p* < 0.001) significantly predicted behavioral intention, whereas the effect of subjective norm was not significant (β = 0.092, t = 1.32, *p* = 0.189) (Table 5, Figure 6). This suggests that the intention to engage in insect-friendly actions is primarily shaped by individuals’ favorable attitudes toward such behaviors and their perceived ability to perform them, rather than by perceived social expectations.

Behavioral intention, in turn, exerted a strong and statistically significant effect on behavior (β = 0.572, t = 14.98, *p* < 0.001), confirming its central role as the immediate antecedent of behavior. Indirect effects further supported this mediation pattern: both attitude (AT → INT → BEH: β = 0.150, t = 3.08, *p* = 0.002) and perceived behavioral control (PBC → INT → BEH: β = 0.173, t = 4.00, *p* < 0.001) showed significant indirect effects on behavior via intention, while the subjective norm → intention → behavior path remained nonsignificant (β = 0.052, *p* = 0.189).

The model explained 37.3% of the variance in behavioral intention (R^2^ = 0.373, *p* < 0.001) and 32.7% of the variance in behavior (R^2^ = 0.327, *p* < 0.001) (Figure 6), indicating moderate explanatory power consistent with typical TPB results. Predictive relevance confirmed that the model had moderate predictive accuracy for behavior (Q^2^ = 0.219) and substantial predictive relevance for behavioral intention (Q^2^ = 0.358).

#### 3.2.4. The Integrated Model VBN + TPB

The integrated model demonstrated high explanatory power, accounting for 62% of the variance in behavioral intention (R^2^ = 0.620, Q^2^ = 0.524) and 35.1% of the variance in behavior (R^2^ = 0.351, Q^2^ = 0.252).

The structural relationships within the VBN pathway were all positive and statistically significant: BV → WW (β = 0.930, *p* < 0.001), WW → MN (β = 0.988, *p* < 0.001), MN → INT (β = 0.516, *p* < 0.001), and INT → BEH (β = 0.593, *p* < 0.001) (Table 6). These results indicate that biospheric values foster an ecological worldview, which activates moral norms that, in turn, drive behavioral intentions and actual engagement.

**Figure 7:**
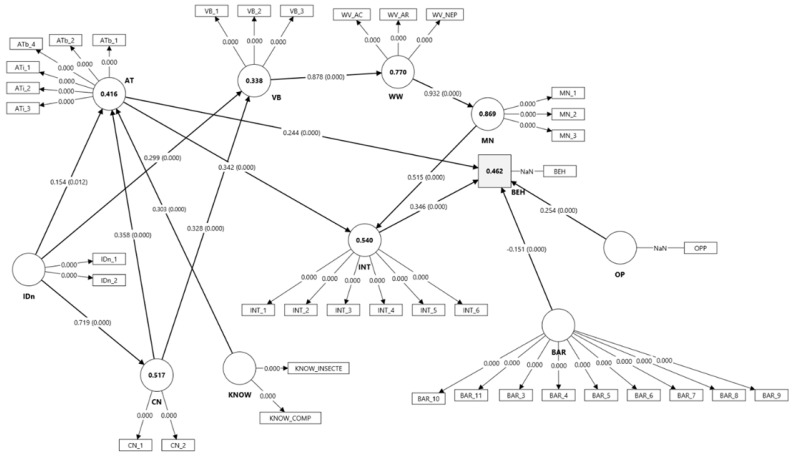
PLS-SEM structural model of the proposed model. (AT—Attitude; SN—Subjective Norms; PBC—Perceived Behavioral Control; INT—Behavioral Intention; BEH—Behavior; BV—Biospheric Values; NEP—Ecological Beliefs; AC—Awareness of Consequences; AR—Ascription of Responsibility; MN—Moral Norms; KNOW—Knowledge about Insects; IDn—Naturalist Identity; CN—Nature Connectedness; BAR—Perceived Barriers; OPP—Perceived Opportunities).

In contrast, the TPB-related predictors of behavioral intention and behavior, attitudes (AT: β = 0.136, *p* < 0.326), subjective norms (NS: β = 0.140, *p* < 0.359), and perceived behavioral control (PBC: β = 0.124, *p* < 0.390), were not statistically significant (Table 6, Figure 7). This pattern suggests that, when moral norms are explicitly incorporated, the traditional TPB predictors become less influential, with moral and value-based motivations dominating the prediction of behavioral intention.

Indirect effects further confirmed this mediating chain. The sequential pathways VB → WW → MN → INT → BEH (β = 0.286, *p* < 0.001) and WW → MN → INT → BEH (β = 0.307, *p* < 0.001) were significant, supporting the idea that the influence of biospheric values on behavior is mediated through worldview, moral norms, and intention. In contrast, the indirect effects involving AT, NS, and PBC were nonsignificant (all *p* > 0.10).

Based on evidence from previous studies suggesting that attitudes can exert a direct influence on behavior, we re-estimated the combined VBN–TPB model by introducing direct paths from Attitude (AT) to Behavior (BEH) while retaining the existing AT → INT connection. In this revised specification, the VBN causal chain remained robust, with all hypothesized links (BV → WW; WW → MN; MN → INT; INT → BEH) remaining positive and statistically significant (Table 6).

Both newly specified paths, AT → BEH and AT → INT, emerged as positive and significant predictors of behavior (AT → BEH: β = 0.253, *p* < 0.001; AT → INT: β = 0.166, *p* < 0.001). The inclusion of these direct effects improved the overall explanatory and predictive performance of the model, increasing the variance explained in behavior from R^2^ = 0.351 (Q^2^ = 0.252) in the previous combined model to R^2^ = 0.372 (Q^2^ = 0.283), while intention also remained well predicted (R^2^ = 0.554; Q^2^ = 0.529).

However, despite its superior explanatory power (Table 7), the combined model revealed that only the VBN-based pathways were statistically significant, while the three main TPB paths did not reach statistical significance, with attitude emerging as the only significant predictor in the final specification. Therefore, H1 is only partially supported by the results.

#### 3.2.5. Final Model

Prior to incorporating the additional variables into the model, a comparison of the three tested models was conducted in order to identify and retain only the statistically significant paths (Table 7). As a result, within the TPB framework, only the attitude construct remained directly linked to behavior and behavioral intention, whereas perceived behavioral control and subjective norm were excluded from the subsequent analyses (Table 8).

**H2.**  *Knowledge*

Knowledge exhibited a strong and consistent positive influence on key psychological determinants of pro-environmental action. It significantly predicted attitudes (β = 0.301, t = 6.794, *p* < 0.001), behavior (β = 0.261, t = 5.909, *p* < 0.001), and perceived behavioral control (β = 0.338, t = 6.464, *p* < 0.001). These results support H2a–H2c, confirming that higher knowledge about insects and their ecological importance enhances both cognitive and behavioral engagement. An additional indirect effect from knowledge through attitudes to behavior was positive and marginally significant (β = 0.052, t = 3.286, *p* = 0.001), indicating that informed individuals are more likely to develop favorable attitudes that translate into pro-environmental intentions and behaviors. Since perceived behavioral control was not retained in the final model, we additionally test whether knowledge directly predicts behavioral intention, consistent with prior evidence that knowledge can foster intention even in the absence of control perceptions [80,81].

**H3.**  *Connection with Nature*

Connection with nature positively influenced both biospheric values (β = 0.328, t = 4.858, *p* < 0.001) and attitudes (β = 0.359, t = 5.911, *p* < 0.001), supporting H3a and H3b. These results align with prior findings [82,83], showing that individuals who feel emotionally connected to nature tend to endorse stronger biospheric values and exhibit more favorable attitudes toward insects and ecological conservation. Indirectly, connection with nature also exerted a significant total effect through the extended VBN pathway (CN → VB → WW → MN → INT → BEH, β = 0.038, t = 3.183, *p* = 0.001), demonstrating that nature connectedness contributes to behavior through both moral–normative and attitudinal channels.

**H4.**  *Naturalist Identity*

Naturalist identity showed a significant positive effect on both biospheric values (β = 0.300, *p* < 0.001) and attitudes (β = 0.154, *p* = 0.011), supporting H4a and H4b. Indirect effects were also observed along the VBN chain (IDn → BV → WW → MN → INT → BEH, β = 0.035, *p* < 0.001), as well as through shorter mediational routes (IDn → BV → WW → MN, β = 0.245, *p* < 0.001; IDn → BV → WW, β = 0.263, *p* < 0.001). A smaller yet significant indirect path through attitudes (IDn → AT → BEH, β = 0.027, *p* = 0.040) further indicated that identity influences both value-driven and attitudinal mechanisms leading to behavior.

**H5.**  *Situational Factors (Barriers and Opportunities)*

Situational conditions exerted meaningful and opposing effects on pro-environmental behavior. Perceived barriers negatively predicted behavior (β = −0.151, *p* < 0.001), supporting H5a–H5b. In contrast, perceived opportunities positively influenced behavioral intention (β = 0.159, t = 4.435, *p* < 0.001) and behavior (β = 0.254, t = 6.169, *p* < 0.001). These findings highlight that external enablers and constraints play a decisive role in facilitating or limiting insect-supportive behaviors.

## 4. Discussion

The present study represents the first attempts to systematically examine human–insect relationships in Romania and contributes to the limited body of literature on this topic internationally. The current study used a comprehensive theoretical model by integrating the TPB and VBN variables, together with additional constructs: knowledge about insects, naturalist identity, nature connectedness, perceived barriers, and perceived opportunities to evaluate the factors shaping individuals’ insect-friendly behaviors.

### 4.1. Knowledge, Attitudes, and Perceived Barriers in Insect Conservation

Findings show a strong link between knowledge and positive attitudes toward insects, consistent with prior research [42,79]. Accurate information appears crucial for shaping positive orientations, especially given the persistence of misconceptions that fuel fear and aversion. Demographic and affective factors also mattered: older participants and men reported more positive attitudes, while emotional connection with nature, naturalist identity, and frequent exposure to nature significantly enhanced conservation concern [82,83,84].

The present findings diverge from the traditional knowledge–action gap described in the environmental psychology literature [78,109], which assumes that individuals possess the necessary knowledge but fail to act upon it. In contrast, the results of this study indicate a more fundamental knowledge deficit: respondents did not demonstrate a clear understanding of what specific actions are required to protect insects. While general awareness of the ecological importance of insects was moderate to high, knowledge of concrete, insect-friendly conservation practices was markedly low. Misconceptions, such as the belief that beekeeping can reverse insect decline or that only two bee species exist, were widespread, reflecting limited exposure to accurate information.

This pattern suggests that, in the Romanian context, insufficient knowledge and limited access to reliable information represent one of several barriers to the adoption of pro-conservation behaviors. While motivational and attitudinal factors play an important role, the lack of clear understanding regarding specific conservation actions appears to further hinder behavioral engagement. In contrast to countries with a long-standing tradition of environmental education and citizen science initiatives [78,110], Romania’s public involvement in biodiversity conservation remains in a formative stage. As a result, the general population may not yet have developed an adequate informational and experiential foundation to consistently translate positive attitudes into responsible actions toward insect protection.

These findings are also influenced by the age profile of the sample, which can be identified as a limitation, predominantly composed of young adults (18–24 years), a generation with limited formal exposure to entomological topics, typically restricted to two biology lessons in the sixth-grade curriculum. Prior research has shown that childhood experiences in nature are crucial for developing ecological awareness and empathy [30]. Therefore, the relatively low knowledge levels observed among younger respondents may reflect both educational limitations and the broader phenomenon of “extinction of experience”, which describes the declining direct interaction with nature among modern youth populations [33,35]. Also, another identified limitation resides in the lack of information about the curricular content and the expertise (in terms of ecological education) of the teaching staff that delivered entomological topics to some of the respondents.

Perceived barriers further reinforced this lack of knowledge, time, and financial resources which were the most frequently reported obstacles, with informational barriers proving as limiting as logistical ones. Importantly, perceived barriers correlated negatively with self-reported conservation behaviors, consistent with prior findings that constraints, real or perceived, can suppress environmental action [87,94,95,111]. Addressing these barriers through accessible, evidence-based communication and practical guidance is thus critical for supporting broader engagement in insect conservation.

### 4.2. Integrating Theoretical Frameworks to Explain Insect Conservation Behavior

Behind such complex behaviors that comprise the protection and conservation of insects there is an entire hierarchical motivational architecture, with factors related to different registers of psychological functioning and not only, factors that have variable predictive values. To this end, the present study verified the predictive value of three explanatory models to identify the most effective vectors of educational influence—the Theory of Planned Behavior (TPB), the Value–Belief–Norm (VBN) and an integrated framework that synthesizes both while adding additional variables: the level of accumulated knowledge, the nature connectedness, the naturalistic identity or the direct contextual variables: barriers and opportunities. The perspectives that the two basic models offer are complementary, The VBN framework captures the deep-seated moral and value-based foundations of ecological engagement; the TPB adds a rational–cognitive layer. While both the VBN and TPB models exhibited significant pathways when analyzed separately, the TPB predictors, attitudes, subjective norms, and perceived behavioral control, lost statistical significance in the combined model. This contrasts with other environmental behaviors, such as water conservation behavior [56], recycling [57,58], energy conservation [61,62,86] or sustainable mobility choices [112] where well-established social expectations and infrastructures reinforce the predictive power of subjective norms and perceived control.

However, when attitudes were modeled to influence behavior both directly and indirectly through intention, they became positive and statistically significant, improving the overall explanatory power of the model. This result aligns with theoretical extensions of the TPB, which suggest that under certain conditions, such as when behaviors are emotionally salient, familiar, or personally relevant, attitudes can directly predict behavior, bypassing the mediating role of intention [48,113]. In this case, attitudes toward insects likely function more as affective evaluations than as purely cognitive judgments, shaped by emotional reactions such as fear, empathy, or fascination. These emotions can either motivate protective behaviors or lead to avoidance and indifference [28,31]. Understanding this affective basis is crucial for designing educational and communication strategies that reshape public perceptions and foster positive engagement with insects.

In the collective imagination, insects, unlike other living species (e.g., plants), are often attributed a series of negative qualities [23,27,28,31], which creates a wide range of emotions and attitudes that cannot be captured by cognitive-rational mechanisms. It is therefore understandable that for protective intentions and behaviors towards insects, the VBN model which focuses on determinations related to emotional, attitudinal, and value registers has a greater predictive value. Making a comparison between the predictive value of moral norms, it is observed that for insects and their protection, internalized moral norms (VBN) (biospheric values, ecological worldview, moral responsibility and personal moral norms) are stronger than subjective norms (social pressure or expectations of others) analyzed by TPB.

In the Romanian context, this pattern may be further reinforced by the limited institutionalization of pro-environmental social norms. Currently, there is no strong social expectation or cultural framework that encourages individuals to take action for insect protection. Similar findings have been observed across cultural contexts, where the influence of social norms on pro-environmental behavior is highly dependent on cultural orientation and the degree of social norm internalization [114]. As social norms often serve as key sources of information and behavioral guidance, defining what is appropriate or expected within a community [115], their absence weakens the normative pressure to act.

In contrast, personal or moral norms, as conceptualized in the VBN framework, are activated when individuals: (a) become aware that their actions have consequences for the well-being of others (awareness of consequences) and (b) accept personal responsibility for those consequences (ascription of responsibility) [16,53]. When both conditions are met, moral norms become strong motivators of behavior, even in the absence of strong social expectations [16]. This distinction may explain why, in domains such as recycling or plastic reduction, where societal expectations and visibility of environmental problems are high, individuals experience both social pressure and moral obligation to act [81,112]. Conversely, the decline of insects is a less visible and less publicly discussed issue, leading to weaker subjective norms and reliance primarily on internalized moral motivations.

Empirical studies on insect conservation psychology confirm that people’s engagement in insect-friendly actions depend not only on ecological knowledge but also on the development of a culture of personal and collective responsibility [116]. In Romania, public perceptions of environmental issues remain fragmented, with awareness often unaccompanied by consistent behavioral norms or civic engagement [117]. Moreover, insects are frequently perceived primarily through their utility to humans, rather than as intrinsic components of ecosystems. Despite their ecological importance, knowledge and public awareness about insect decline remain limited, and there is not yet a widespread cultural or normative framework that encourages insect-friendly behaviors [116,118]. Consequently, moral norms, rather than social expectations, played the dominant role in predicting responsible behavior in this study.

Beyond the core VBN–TPB integration, the inclusion of additional psychological and contextual variables, knowledge, connection with nature, naturalist identity, barriers, and opportunities, provided deeper insight into the mechanisms that shape responsible behavior toward insects. These results are in line with previous works [75,76,78]. Knowledge emerged as a key determinant, positively influencing both attitudes and perceived behavioral control, as well as directly predicting behavior [79,80,81]. This highlights the importance of educational and informational interventions, as awareness remains a prerequisite for activating moral and behavioral engagement. Feeling connected to nature and identifying with naturalist roles were associated with stronger biospheric values and more positive attitudes, a pattern similar to reports that tie emotion and identity to pro-environmental action [32,84,85,92,93]. Situational factors such as barriers and opportunities also shaped engagement, barriers reduced behavioral control and action, while opportunities facilitated behavioral translation. Taken together, the final model highlights the dominant role of the VBN pathway, where connection with nature, biospheric values, worldviews, and moral norms jointly explain intention and behavior. At the same time, attitudinal and contextual influences (knowledge, opportunities, barriers) add complementary predictive power, ensuring that the model integrates both deep-seated normative motivations and situational enablers of pro-environmental action.

### 4.3. Practical and Educational Implications

Translated into educational practice, the results illustrated in Figure 7 identify not only the determinants of protective behaviors toward insects but also a hierarchy of educational interventions capable of strengthening them. The integrated TPB–VBN model suggests that effective conservation education should begin with affective and motivational activation before introducing cognitive or informational components. Moral and ethical engagement, empathy, and emotional connection with nature should precede knowledge acquisition in order to counteract the negative emotional perceptions often associated with insects.

#### 4.3.1. Early Childhood Interventions and Role Modeling

Nature connectedness and naturalist identity are often established during early childhood through direct and sustained interaction with natural environments. Experiences in memorable outdoor settings help children develop emotional bonds, positive values, and a lifelong commitment to the environment [32,119]. Unstructured play in natural spaces enhances creativity, problem-solving, resilience, and emotional well-being [120], while access to green areas encourages exploration and curiosity [119]. Adult role models, parents, grandparents, and teachers, reinforce these experiences by modeling empathy, respect, and care for living organisms. Such formative encounters lay the foundation for enduring pro-environmental values and behaviors that persist into adulthood [32].

#### 4.3.2. Knowledge Integration and Cognitive Reinforcement

After affective and moral dimensions have been activated, educational programs should focus on reinforcing ecological literacy. Lessons on the ecological roles of insect pollination, decomposition, and ecosystem balance, can be integrated across disciplines such as biology, geography, and civic education. Inquiry-based and experiential methods, including mini-research projects or building insect habitats, help translate curiosity into understanding and sustained engagement.

#### 4.3.3. Community Engagement and Opportunity Creation

At the community level, initiatives such as pollinator-friendly gardens, urban biodiversity zones, and intergenerational conservation projects provide accessible opportunities for participation and reduce perceived barriers to action. Collaborative programs involving schools, NGOs, and local authorities can help institutionalize pro-environmental norms and increase collective efficacy. Highlighting positive social models: teachers, gardeners, or citizens actively engaged in insect protection, can strengthen emerging social expectations in relation to an integrative ecological mindset.

#### 4.3.4. Communication and Cultural Change

Promoting behavioral change toward insect conservation requires not only the dissemination of ecological knowledge but also the transformation of underlying cultural narratives and emotional associations. Insects have traditionally occupied ambivalent positions within human culture, symbolizing both fascination and fear. Across most cultures, positive and negative representations of insects coexist, often associated with specific species and the nature of human contact with them. Insects that play visible ecological or symbolic roles, such as bees or ladybugs, are typically linked to values like diligence, luck, or renewal, whereas those encountered in domestic or urban settings, such as flies, mosquitoes, or cockroaches, tend to evoke discomfort, disgust, or fear. These contrasting perceptions are reinforced through everyday experience, popular media, mythology, and folklore, shaping the ambivalent emotional landscape that characterizes human–insect relationships.

In the Romanian cultural context, ‘*useful insects*’ such as bees and ants are often associated with virtues like diligence, solidarity, and natural order, whereas species perceived as harmful, mosquitoes, flies, or cockroaches, evoke feelings of repulsion and fear, being symbolically tied to dirt or disease [121]. The persistence of these representations, transmitted across generations through proverbs, folktales, and traditional practices, contributes to ambivalent attitudes toward insects that combine appreciation with avoidance. Communication strategies should therefore aim to reframe these cultural meanings by emphasizing the ecological indispensability, aesthetic diversity, and cultural significance of insects.

Integrating scientific information with emotionally engaging storytelling can foster empathy and reshape collective perceptions. Art, media, and community-based initiatives, such as educational exhibitions, urban biodiversity festivals, or artistic projects celebrating pollinators, can make insects visible within public spaces and collective imagination. In Romania, where ecological education and insect conservation are still emerging fields, communication efforts may benefit from connecting ecological messages with culturally familiar and positive symbols, thereby fostering acceptance and identification.

Cultural perceptions also play an important role in shaping attitudes toward entomophagy. While the present study did not address edible insect consumption, it is important to acknowledge that cultural representations, whether of insects as food, symbols, or ecological partners, can influence public openness to sustainability-related behaviors. Positive cultural reframing could thus contribute not only to conservation-oriented attitudes but also to the gradual normalization of alternative, ecologically responsible practices, including edible insect acceptance, in contexts where such practices are novel.

#### 4.3.5. Limitation of the Study

A key limitation of this study concerns the sociodemographic composition of the sample, which was not evenly distributed across gender and residential categories. Women were substantially overrepresented (73.7%), while men accounted for 26.3% of participants. Although the difference in attitudes toward insects between women (M = 4.60, SD = 1.40) and men (M = 4.99, SD = 1.25) was statistically significant (t(337) = −2.44, *p* = 0.016), the effect size was small (d = 0.29), indicating that both groups hold generally positive views of insects. However, because women, who expressed slightly less positive attitudes, were numerically dominant, the overall mean (M = 4.70, SD = 1.37) may slightly underestimate the general level of positivity that would be expected in a more gender-balanced sample.

The use of a convenience sampling method further contributed to a predominance of participants residing in urban areas. Prior research has shown that urban residents often exhibit less favorable attitudes toward insects than rural inhabitants [122], potentially influencing the overall averages. In this study, however, no significant differences were observed between respondents from urban and rural settings. This lack of difference may be explained by the age structure of the sample, largely composed of young adults aged 18–24, for whom direct contact with nature has been increasingly replaced by digital interaction, a process described as the “extinction of experience.” In Romania, where internet access and digital infrastructure are highly developed nationwide, young people from both urban and rural areas are equally exposed to technology, which may mitigate traditional disparities in attitudes toward nature and insects.

While these sociodemographic imbalances limit the generalizability of the results to the entire Romanian population, they nonetheless provide a valuable foundation for understanding behavioral determinants of insect conservation. Moreover, they highlight key demographic segments that can serve as target groups for awareness campaigns and educational initiatives tailored to the Romanian socio-cultural context.

## 5. Conclusions

This study provides one of the first empirical examinations of responsible behavior toward insects through an integrative Value–Belief–Norm (VBN) and Theory of Planned Behavior (TPB) framework. Conducted in Romania, it extends pro-environmental behavior research by demonstrating that moral and value-based predictors remain central even when combined with rational–cognitive components. Knowledge, connection with nature, and naturalistic identity further reinforced positive attitudes and moral engagement, while situational factors such as perceived barriers and opportunities influenced the translation of intention into action. These results emphasize that fostering insect-friendly behavior requires more than information transfer. In our opinion, this process demands emotional and moral activation supported by experiential and community-based learning. By combining cognitive, affective, and contextual dimensions, the proposed model offers a comprehensive integrative framework for designing educational and communication strategies capable of transforming awareness into lasting pro-environmental commitment and contributing to the broader goal of biodiversity conservation.

## Figures and Tables

**Table 1:** Sample questionnaire items across the measured constructs. This table displays illustrative items included in the survey instrument. The full set of items is available in the Supplementary Materials, Document S1.

Section	Variable	Item	Scale/Response Options
Demographic Data	Gender	What is your gender?	Female/Male/Other/Prefer not to say
	Age	In which age category do you fall?	18–24; 25–34; 35–44; 45–54; 55–64; Over 65
	Employment status	What is your current employment situation?	Student; Employed; Unemployed; Retired; Other
	Field of activity	In which field do you currently work or have you worked?	Open-ended/short answer
TPB	Attitudes toward insects	Insects are very important to me.	Likert (1–7): (1) Strongly disagree–(7) Strongly agree
		I find insects very interesting.	
		I care about doing things for insects.	
		I find insects disgusting.	
	Attitudes toward insect-related behavior	My actions at home can help insects.	Likert (1–7)
		My actions at work can help insects.	
		Nothing I do for insects will make a difference.	
	Subjective norms	Most people who are important to me think I should protect insects	Likert (1–7)
		Most people who I respect and admire engage in insects’ conservation actions.	
	Perceived behavioral control	I am confident that I can do things to help insects.	Likert (1–7)
		It is difficult for me to help insects at home.	
		I have the knowledge and skills to behave eco-friendly towards insects.	
	Intention	I intend to create a pollinator-friendly garden to support insect populations.	Likert (1–7)
		I intend to plant native flowering species to provide food and shelter for insects.	
VBN	Biospheric values	Respect for the planet and preventing pollution are important to me.	Likert (1–7)
		Unity with nature is a priority.	
	New Ecological Paradigm	Plants and animals have as much right to live as humans.	Likert (1–7)
		Humans are seriously abusing the environment.	
	Awareness of consequences	Protection of the environment benefits us all.	Likert (1–7)
		The environmental damage we cause here affects people all over the world.	
		Environmental protection will mean a better world for me and my children.	
	Ascription of responsibility	Every member of the public should accept responsibility for the environment.	Likert (1–7)
		The authorities are responsible for the environment	
	Moral norm	I feel morally obligated to engage in environmental behaviors regardless of what others are doing	Likert (1–7)
		I feel behaving in an eco-friendly way is the right thing to do.	
Knowledge	Knowledge about insects	Insects are an essential part of nature	True/False/Not sure
		There are 2 species of bee	
		Flies and beetles can be pollinators.	
	Knowledge about insect-friendly actions	Providing more wildflowers will help to reverse insect declines	True/False/Not sure
		Keeping honeybees will help to reverse insect declines	
		Mowing lawns and parks less often will help to reverse insect declines	
Perceived barriers		I have limited space (e.g., I don’t have a garden, etc.).	Likert (1–7)
Self-reported behavior		I maintain unmown areas in the green spaces I own (gardens, meadows, or similar).	Select all that apply
		I avoid using insecticides as much as possible.	
		On the land I manage, I leave areas with wild vegetation.	
Naturalist identity		Protecting nature is an important part of who I am.	Likert (1–7)
		I consider myself a person who takes care of nature.	
Nature connectedness		I feel that I am part of nature.	Likert (1–7)
		I am not the kind of person who is interested in nature.	
Perceived opportunities		I own/manage a garden	Select all that apply
		I am a beekeeper	

**Table 2:** Reliability and Validity Assessment of the VBN Model. CR—composite reliability; AVE—average variance extracted; All outer loadings are significant at *p* = 0.001 level. (INT—behavioral intention; MN—moral norms; VB—biospheric values; WW—Worldview (AC—awareness of consequences; AR—ascription of personal responsibility; NEP—New Environmental Paradigm, ecological beliefs)).

	Outer Loadings	Cronbach’s Alpha	CR	AVE
INT		0.922	0.926	0.719
INT_1	0.873 ***			
INT_2	0.852 ***			
INT_3	0.890 ***			
INT_4	0.804 ***			
INT_5	0.835 ***			
INT_6	0.831 ***			
MN		0.945	0.948	0.901
MN_1	0.917 ***			
MN_2	0.964 ***			
MN_3	0.966 ***			
VB		0.945	0.952	0.901
VB_1	0.958 ***			
VB_2	0.926 ***			
VB_3	0.964 ***			
WW		0.938	0.939	0.891
WW_AC	0.953 ***			
WW_AR	0.944 ***			
WW_NEP	0.934 ***			

*** → *p* < 0.001.

**Table 3:** Reliability and Validity Assessment of the TPB Model. CR—composite reliability; AVE—average variance extracted; All outer loadings are significant at *p* = 0.001 level. (INT—behavioral intention; AT—attitudes; ATb—attitudes toward insect-related behavior; ATi—attitude toward insects; SN—subjective norms; PBC—perceived behavioral control).

	Outer Loadings	Cronbach’s Alpha	CR	AVE
INT		0.922	0.939	0.719
INT_1	0.862 ***			
INT_2	0.832 ***			
INT_3	0.877 ***			
INT_4	0.823 ***			
INT_5	0.855 ***			
INT_6	0.839 ***			
AT		0.931	0.946	0.745
ATb_1	0.882 ***			
ATb_2	0.834 ***			
ATb_4	0.808 ***			
ATi_1	0.893 ***			
ATi_2	0.847 ***			
ATi_3	0.911 ***			
SN		0.751	0.849	0.654
SN_1	0.859 ***			
SN_2	0.718 ***			
SN_3	0.841 ***			
PBC		0.886	0.921	0.745
PBC_1	0.841 ***			
PBC_4	0.883 ***			
PBC_5	0.894 ***			
PBC_6	0.834 ***			

*** → *p* < 0.001.

**Table 4:** Path coefficients and mediation effects in the VBN Model.

	Coefficients	M	STDEV	T	*p* Values
Direct paths					
INT → BEH	0.568	0.569	0.039	14.721	0.000
MN → INT	0.672	0.671	0.037	18.382	0.000
VB → WW	0.879	0.879	0.017	50.690	0.000
WW → MN	0.932	0.932	0.009	101.494	0.000
Specific indirect effects					
MN → INT → BEH	0.382	0.382	0.033	11.633	0.000
VB → WW → MN	0.819	0.819	0.021	38.175	0.000
WW → MN → INT → BEH	0.356	0.356	0.032	11.080	0.000
WW → MN → INT	0.626	0.626	0.038	16.460	0.000
VB → WW → MN → INT	0.550	0.550	0.039	14.033	0.000
VB → WW → MN → INT → BEH	0.313	0.313	0.031	10.061	0.000

**Table 5:** Path coefficients and mediation effects in the TPB model.

	Coefficients	M	STDEV	T	*p* Values
Direct paths					
AT → INT	0.262	0.263	0.081	3.250	0.001
INT → BEH	0.572	0.573	0.038	14.981	0.000
PBC → INT	0.303	0.303	0.073	4.158	0.000
SN → INT	0.092	0.093	0.070	1.315	0.189
Specific indirect effects					
PBC → INT → BEH	0.173	0.174	0.043	4.001	0.000
AT → INT → BEH	0.150	0.151	0.049	3.079	0.002
SN → INT → BEH	0.052	0.053	0.040	1.315	0.189

**Table 6:** Path coefficients and mediation effects in the integrated model VBN + TPB.

	Coefficients	M	STDEV	T	*p* Values
Direct paths					
AT → BEH	0.260	0.261	0.047	5.563	0.000
AT → INT	0.168	0.169	0.075	2.246	0.025
INT → BEH	0.420	0.420	0.051	8.272	0.000
MN → INT	0.490	0.488	0.054	9.001	0.000
NS → INT	0.105	0.108	0.068	1.556	0.120
PBC → INT	0.131	0.131	0.071	1.851	0.064
VB → WW	0.879	0.879	0.017	50.690	0.000
WW → MN	0.932	0.932	0.009	101.498	0.000
Specific indirect effects					
MN → INT → BEH	0.311	0.311	0.040	7.726	0.000
VB → WW → MN → INT	0.482	0.482	0.060	7.995	0.000
VB → WW → MN	0.919	0.919	0.017	52.797	0.000
PBC → INT → BEH	0.074	0.069	0.085	0.864	0.388
SN → INT → BEH	0.083	0.089	0.091	0.914	0.361
WW → MN → INT	0.518	0.518	0.063	8.251	0.000
VB → WW → MN → INT → BEH	0.286	0.286	0.039	7.383	0.000
WW → MN → INT → BEH	0.307	0.307	0.040	7.631	0.000
AT → INT → BEH	0.081	0.081	0.083	0.971	0.000

**Table 7:** Comparison between the proposed models and the original TPB and VBN models.

	VBN		TPB		VBN + TPB (Classic)		VBN + TPB (Direct Path AT → BEH)		Final Model	
	R^2^	Q^2^	R^2^	Q^2^	R^2^	Q^2^	R^2^	Q^2^	R^2^	Q^2^
BEH	0.323	0.168	0.327	0.219	0.351	0.251	0.372	0.283	0.460	0.340
INT	0.451	0.444	0.373	0.358	0.554	0.529	0.554	0.529	0.554	0.248

**Table 8:** Path coefficients and mediation effects in the final model.

	Coefficients	M	STDEV	T	*p* Values
Direct paths					
AT → BEH	0.244	0.243	0.049	4.990	0.000
AT → INT	0.342	0.343	0.049	6.938	0.000
BAR → BEH	−0.151	−0.156	0.042	3.562	0.000
CN → AT	0.358	0.358	0.061	5.879	0.000
CN → VB	0.328	0.329	0.068	4.844	0.000
IDn → AT	0.154	0.154	0.061	2.523	0.012
IDn → CN	0.719	0.719	0.033	21.782	0.000
IDn → VB	0.299	0.297	0.065	4.621	0.000
INT → BEH	0.346	0.345	0.053	6.553	0.000
KNOW → AT	0.303	0.306	0.044	6.903	0.000
MN → INT	0.515	0.513	0.050	10.268	0.000
OP → BEH	0.254	0.253	0.041	6.169	0.000
VB → WW	0.878	0.877	0.018	49.722	0.000
WW → MN	0.932	0.932	0.009	101.492	0.000
Specific indirect effects					
IDn → CN → AT	0.257	0.258	0.047	5.486	0.000
CN → VB → WW → MN → INT	0.138	0.138	0.033	4.236	0.000
CN → VB → WW	0.288	0.289	0.060	4.784	0.000
MN → INT → BEH	0.178	0.177	0.031	5.747	0.000
IDn → VB → WW	0.262	0.261	0.057	4.589	0.000
WW → MN → INT	0.480	0.478	0.049	9.766	0.000
KNOW → AT → INT → BEH	0.036	0.036	0.009	3.854	0.000
CN → VB → WW → MN → INT → BEH	0.048	0.048	0.014	3.416	0.001
IDn → CN → VB	0.236	0.237	0.052	4.564	0.000
WW → MN → INT → BEH	0.166	0.165	0.029	5.640	0.000
IDn → CN → AT → INT	0.088	0.088	0.020	4.291	0.000
IDn → CN → AT → BEH	0.063	0.063	0.017	3.783	0.000
VB → WW → MN → INT	0.421	0.420	0.047	9.040	0.000
IDn → VB → WW → MN → INT	0.126	0.125	0.031	4.089	0.000
IDn → VB → WW → MN	0.244	0.243	0.054	4.561	0.000
IDn → AT → INT → BEH	0.018	0.018	0.008	2.162	0.031
IDn → CN → VB → WW → MN → INT	0.099	0.100	0.025	3.942	0.000
IDn → VB → WW → MN → INT → BEH	0.044	0.043	0.012	3.682	0.000
CN → AT → BEH	0.087	0.087	0.022	3.919	0.000
IDn → AT → BEH	0.038	0.037	0.017	2.260	0.024
VB → WW → MN	0.818	0.817	0.022	37.640	0.000
CN → AT → INT	0.122	0.123	0.028	4.379	0.000
KNOW → AT → BEH	0.074	0.075	0.020	3.718	0.000
IDn → AT → INT	0.053	0.053	0.022	2.340	0.019
IDn → CN → VB → WW → MN	0.193	0.194	0.043	4.444	0.000
KNOW → AT → INT	0.104	0.105	0.022	4.743	0.000
CN → VB → WW → MN	0.269	0.269	0.057	4.748	0.000
IDn → CN → VB → WW	0.207	0.208	0.046	4.487	0.000
IDn → CN → AT → INT → BEH	0.030	0.031	0.009	3.375	0.001
CN → AT → INT → BEH	0.042	0.043	0.012	3.456	0.001
VB → WW → MN → INT → BEH	0.146	0.145	0.027	5.399	0.000
AT → INT → BEH	0.118	0.119	0.026	4.571	0.000
IDn → CN → VB → WW → MN → INT → BEH	0.034	0.035	0.011	3.221	0.001
IDn → CN → AT	0.257	0.258	0.047	5.486	0.000

## Data Availability

The original contributions presented in this study are included in the article/Supplementary Material. Further inquiries can be directed to the corresponding authors.

## Supplementary Materials

The following supporting information can be downloaded at: https://www.mdpi.com/article/10.3390/insects16121274/s1, Document S1: Complete survey instrument based on the Value–Belief–Norm Theory and the Theory of Planned Behavior; Table S1: Outer Weights of the Barriers Construct (items BAR_1 and BAR_2 removed from the Final Model); Table S2: Heterotrait–Monotrait Ratio Values in the Classic VBN Model (INT—behavioral intention; MN—moral norms; VB—biospheric values; AC—awareness of consequences; AR—ascription of personal responsibility; NEP—New Environmental Paradigm, ecological beliefs); Table S3: Outer loadings in the Classic VBN Model (INT—behavioral intention; MN—moral norms; VB—biospheric values; AC—awareness of consequences; AR—ascription of personal responsibility; NEP—New Environmental Paradigm, ecological beliefs); Table S4: Outer loading in the VBN Model with Worldview (WW) (INT- behavioral intention; MN—moral norms; VB—biospheric values; AC—awareness of consequences; AR—ascription of personal responsibility; NEP—New Environmental Paradigm, ecological beliefs); Table S5a: Outer Loading Values in the Initial TPB Model, with attitude split into two constructs: attitude toward insects (Ati) and attitude toward insect-related behaviors (Atb); Table S5b: Heterotrait-monotrait ratio in the Initial TPB Model, with attitude split into two constructs: attitude toward insects (Ati) and attitude toward insect-related behaviors (Atb); Table S6: Reliability and Validity Assessment of the Final Model.
