# Supplementary material for: From Values to Action: An Integrative Explanatory Framework for Insect Conservation Intentions and Behavior
Source: Insects. 2025 Dec 15;16(12):1274. doi: 10.3390/insects16121274 (PMC12733544; doi:10.3390/insects16121274)
Supplement: Supplementary file 1 [file insects-16-01274-s001.zip › S1. questionnaire.pdf]

S1. Complete Survey Instrument Based on the Value–Belief–Norm Theory and the Theory of Planned Behavior

Demographic Variables

1. What is your gender?   Female / Male / Other / Prefer not to answer
2. What is your age category?
   • 18–24   • 25–34   • 35–44   • 45–54   • 55–64   • 65+
3. What is your current employment status?
   Student / Employed / Unemployed / Retired / Other
4. Which of the following best describes the place where you live?
   • Urban   • Rural
5. What is your monthly income?
6. What is your highest level of completed education?

Section A – Value–Belief–Norm Theory (adapted from Gkargkavouzi et al., 2019)
Response scale for all items below:
(1) Strongly disagree – (7) Strongly agree

A.1. Biospheric Values

1. Respecting the planet and preventing pollution are important to me.
2. Feeling connected to nature is a priority for me.
3. Protecting the environment is particularly significant to me.

A.2. New Ecological Paradigm

1. Plants and animals have the same right to life as humans.
2. Humans seriously abuse the environment.
3. The balance of nature is delicate and easily disrupted.
4. Despite our special abilities, humans are still subject to the laws of nature.
5. The Earth has enough resources; we just need to learn how to use them wisely.

A.3. Awareness of Consequences

1. Everyone benefits from protecting nature.
2. Thousands of species will go extinct in the next decade.
3. What is said about climate change is exaggerated. *(reverse item)*
4. Environmental protection benefits my health.
5. Local environmental destruction affects people worldwide.
6. Protecting the environment means a better future for me and my children.

A.4. Ascription of Responsibility

1. Every member of the community should take responsibility for the environment.
2. The authorities are responsible for environmental protection.
3. I am not concerned about the environment. *(reverse item)*

A.5. Moral Norm

1. I feel morally obliged to behave in an environmentally friendly way regardless of what others do.
2. I feel that behaving ecologically is the right thing to do.
3. I feel good about myself when I behave in an environmentally friendly manner.

A.6. Behavioral Intention

1. I intend to engage in environmentally protective behaviors.
2. I plan to avoid wasting natural resources in the coming months.
3. I will try to reduce my carbon footprint in the coming months.
4. I intend to create a pollinator-friendly garden to support insect populations.
5. I intend to plant native flowering species to provide food and shelter for insects.
6. I will reduce light pollution to support nocturnal insect species.

Section B – Theory of Planned Behavior (adapted from Knapp et al., 2021)
Response scale: (1) Strongly disagree – (7) Strongly agree
B.1. Attitudes Toward Insects

1. Insects are very important to me.
2. I find insects to be very interesting.
3. I care about doing things that help insects.
4. When I see an insect, I have an immediate fear reaction. *(reverse item)*
5. I find insects disgusting. *(reverse item)*
6. There are harmful insects and beneficial insects.

B.2. Attitudes Toward Pro-Insect Behavior

1. My actions at home can help insects.
2. My actions at work can help insects.
3. Nothing I do for insects will make a difference. *(reverse item)*
4. I behave in a considerate way toward insects.

B.3. Subjective Norms

1. We should do more to protect insects.
2. Many people who are important to me think I should protect insects.
3. Many people I respect and admire are involved in insect conservation actions.

B.4. Perceived behavioral control

1. I am confident that I can do things to help insects.
2. It is difficult for me to help insects at home. *(reverse item)*
3. It is difficult for me to help insects at work. *(reverse item)*
4. I have the knowledge and skills needed to behave responsibly toward insects.

5. I believe I am capable of adopting ecologically responsible behaviors toward insects.
6. I find it easy to behave in an environmentally friendly way.

Section C – Self-Reported Environmental Behavior

(Multiple choice – select all that apply)

1. I usually keep wild flowering plants (e.g., dandelions) in my garden, on my land, or in similar areas.
2. I usually prefer to recycle objects rather than throw them away.
3. I plant wild plant species in my garden that are beneficial for insects.
4. I have planted tree or shrub species with nectar-rich flowers (fruit trees, hawthorn, blackthorn, etc.).
5. I have created a wildflower meadow / patch of wildflowers / narrow strips of wildflowers in my garden or something similar.
6. During the summer, in the green areas I manage, I mow the grass rarely — once a month or once every two months.
7. I avoid using herbicides as much as possible.
8. I avoid using insecticides as much as possible.
9. I provide artificial shelters for pollinators, e.g., bee hotels.
10. I have created areas of bare soil for ground-nesting bees.
11. I keep beehives or allow beekeepers to place hives on my property.
12. I have signed a "save the bees" petition.
13. I encourage other people to protect nature.
14. I am a volunteer in an organization involved in nature conservation.
15. I usually buy seasonal, locally grown, and organic food.
16. Whenever possible, I choose to walk or cycle instead of using the car.
17. I maintain unmown areas in the green spaces I own (gardens, meadows, or similar).
18. On the land I manage, I leave areas with wild vegetation.
19. I provide bird feeders.
20. I usually buy eco-friendly products and brands.
21. I have given sugar water to exhausted bees.
22. I have signed a nature conservation petition or participated in a conservation campaign.
23. I am a member of an environmental or nature conservation organization.
24. I do not do any of the above.

Section D – Additional Constructs (adapted from Knapp et al., 2021)

D.1. Perceived Barriers

(scale 1–7 as above)

1. I am already doing enough.
2. I am not physically able.

3. I have limited space (e.g., no garden).
4. I do not have enough time.
5. I do not have enough money.
6. I lack knowledge about how to help.
7. I do not have access to information on how to help.
8. Spaces created for insects would look messy; I would receive complaints.
9. I might get stung.
10. Insect conservation conflicts with other land-use goals.
11. Insects are not important to me.

D.2. Opportunities (Yes/No)

1. I own/lease land that I manage
2. I own/manage agricultural land
3. I own/manage a garden
4. I manage landscaped green spaces (e.g., public parks)
5. I am a beekeeper
6. I own a business with ecologically managed outdoor space
7. None of the above

D.3. Naturalist identity

1. I see myself as someone who cares about nature.
2. Protecting nature is an important part of who I am.

D.4. Connectedness to Nature

1. Being in nature makes me very happy.
2. Spending time in nature is NOT important to me. *(reverse item)*
3. I feel that I am part of nature.
4. I am not the kind of person who is interested in nature. *(reverse item)*

D.5. Knowledge

Knowledge About Insects *(True / False / Not sure)*

1. Insects are an essential part of nature.
2. There are only two species of bees.
3. Flies and beetles can be pollinators.
4. All of our food depends on animal pollination.
5. Some pollinators are in decline.
6. Bees are threatened with extinction.

Knowledge About Pro-Insect Actions *(True / False / Not sure)*

1. Planting wild native flowers in private and public gardens will help reverse insect decline.
2. Beekeeping will help reverse insect decline.
3. Mowing lawns and parks less frequently will help reverse insect decline.
4. Giving sugar water to exhausted bees will help reverse insect decline.
5. Reducing pesticide use will help reverse insect decline.
