# Supplementary material for: From Values to Action: An Integrative Explanatory Framework for Insect Conservation Intentions and Behavior
Source: Insects. 2025 Dec 15;16(12):1274. doi: 10.3390/insects16121274 (PMC12733544; doi:10.3390/insects16121274)
Supplement: Supplementary file 1 [file insects-16-01274-s001.zip › Tables S1-S6.pdf]

Table S1. Outer Weights of the Barriers Construct (items BAR_1 and BAR_2 removed from the Final Model)

	Outer Weights	P values
**BAR_1 <- bar**	-0.003	0.945
**BAR_2 <- bar**	0.050	0.185
**BAR_3 <- bar**	0.094	0.008
**BAR_4 <- bar**	0.132	0.000
**BAR_5 <- bar**	0.076	0.043
**BAR_6 <- bar**	0.264	0.000
**BAR_7 <- bar**	0.235	0.000
**BAR_8 <- bar**	0.108	0.000
**BAR_9 <- bar**	0.215	0.000
**BAR_10 <- bar**	0.132	0.000
**BAR_11 <- bar**	0.232	0.000

Table S2. Heterotrait–Monotrait Ratio Values in the Classic VBN Model (INT- behavioral intention; MN - moral norms; VB - biospheric values; AC - awareness of consequences; AR - ascription of personal responsibility; NEP - New Environmental Paradigm, ecological beliefs)

	Coefficients	M	2.5%	97.5%
AR <-> AC	1.120	1.124	1.057	1.199
BEH <-> AC	0.388	0.388	0.293	0.475
BEH <-> AR	0.563	0.564	0.444	0.673
MN <-> AC	0.957	0.956	0.921	0.985
MN <-> AR	1.078	1.079	1.031	1.135
MN <-> BEH	0.414	0.415	0.321	0.498
NEP <-> AC	0.937	0.936	0.895	0.970
NEP <-> AR	1.024	1.025	0.962	1.092
NEP <-> BEH	0.392	0.393	0.300	0.477
NEP <-> MN	0.925	0.925	0.885	0.959
VB <-> AC	0.876	0.876	0.822	0.923
VB <-> AR	0.798	0.999	0.942	1.058
VB <-> BEH	0.439	0.440	0.355	0.515
VB <-> MN	0.908	0.908	0.857	0.951
VB <-> NEP	0.924	0.924	0.880	0.959

Table S3. Outer loadings in the Classic VBN Model (INT- behavioral intention; MN - moral norms; VB - biospheric values; AC - awareness of consequences; AR - ascription of personal responsibility; NEP - New Environmental Paradigm, ecological beliefs)

	Coefficients	M	STDEV	T	P values
AC_1 <- AC	0.940	0.939	0.015	61.877	0.000
AC_2 <- AC	0.758	0.758	0.034	22.117	0.000
AC_3 <- AC	0.149	0.152	0.056	2.684	0.007
AC_4 <- AC	0.912	0.911	0.019	46.890	0.000
AC_5 <- AC	0.873	0.873	0.031	28.196	0.000

AC_6 <- AC	0.926	0.925	0.016	56.196	0.000
AR_1 <- AR	0.933	0.933	0.014	65.541	0.000
AR_2 <- AR	0.603	0.603	0.052	11.586	0.000
AR_3 <- AR	0.315	0.319	0.048	6.512	0.000
BEH <- BEH	1.000	1.000	0.000	n/a	n/a
MN_1 <- MN	0.895	0.894	0.026	34.657	0.000
MN_2 <- MN	0.924	0.924	0.014	66.204	0.000
MN_3 <- MN	0.951	0.950	0.013	72.262	0.000
NEP_1 <- NEP	0.787	0.787	0.034	23.096	0.000
NEP_2 <- NEP	0.835	0.836	0.037	22.383	0.000
NEP_3 <- NEP	0.883	0.882	0.026	33.486	0.000
NEP_4 <- NEP	0.801	0.801	0.036	22.037	0.000
VB_1 <- VB	0.983	0.982	0.014	70.949	0.000
VB_2 <- VB	0.861	0.862	0.028	31.205	0.000
VB_3 <- VB	0.924	0.924	0.017	53.897	0.000

Table S4. Outer loading in the VBN Model with Worldview (WW) (INT- behavioral intention; MN - moral norms; VB - biospheric values; AC - awareness of consequences; AR - ascription of personal responsibility; NEP - New Environmental Paradigm, ecological beliefs)

	Coefficients	M	STDEV	T	P values
BEH <- BEH	1.000	1.000	0.000	n/a	n/a
INT_1 <- INT	0.873	0.873	0.017	52.637	0.000
INT_2 <- INT	0.852	0.852	0.017	50.964	0.000
INT_3 <- INT	0.890	0.890	0.013	67.471	0.000
INT_4 <- INT	0.804	0.803	0.023	35.457	0.000
INT_5 <- INT	0.835	0.835	0.020	42.802	0.000
INT_6 <- INT	0.831	0.831	0.021	39.283	0.000
MN_1 <- MN	0.917	0.917	0.017	52.627	0.000
MN_2 <- MN	0.964	0.964	0.006	175.174	0.000
MN_3 <- MN	0.966	0.966	0.005	179.793	0.000
VB_1 <- VB	0.958	0.958	0.006	158.117	0.000
VB_2 <- VB	0.926	0.926	0.017	55.334	0.000
VB_3 <- VB	0.964	0.964	0.006	171.287	0.000
WV_AC <- WW	0.953	0.953	0.007	130.897	0.000
WV_AR <- WW	0.944	0.943	0.008	118.449	0.000
WV_NEP <- WW	0.934	0.934	0.010	97.246	0.000

Table S5a. Outer Loading Values in the Initial TPB Model, with attitude split into two constructs: attitude toward insects (Ati) and attitude toward insect-related behaviors (Atb)

	Outer loadings
ATb_1 <- ATb	0.848
ATb_2 <- ATb	0.725
ATb_3 <- ATb	0.399
ATb_4 <- ATb	0.826

ATi_1 <- ATi	0.925
ATi_2 <- ATi	0.830
ATi_3 <- ATi	0.933
ATi_4 <- ATi	0.517
ATi_5 <- ATi	0.478
ATi_6 <- ATi	0.054
BEH <- BEH	1.000
INT_1 <- INT	0.860
INT_2 <- INT	0.748
INT_3 <- INT	0.795
INT_4 <- INT	0.799
INT_5 <- INT	0.870
INT_6 <- INT	0.806
NS_1 <- SN	0.940
NS_2 <- SN	0.840
NS_3 <- SN	0.828
PBC_1 <- PBC	0.815
PBC_4 <- PBC	0.820
PBC_5 <- PBC	0.826
PBC_6 <- PBC	0.789

Table S5b. Heterotrait-monotrait ratio in the Initial TPB Model, with attitude split into two constructs: attitude toward insects (Ati) and attitude toward insect-related behaviors (Atb).

	HTMT
ATi <-> ATb	0.888
BEH <-> ATb	0.565
BEH <-> ATi	0.563
INT <-> ATb	0.647
INT <-> ATi	0.573
INT <-> BEH	0.594
PBC <-> ATb	0.828
PBC <-> ATi	0.766
PBC <-> BEH	0.477
PBC <-> INT	0.642
SN <-> ATb	0.850
SN <-> ATi	0.764
SN <-> BEH	0.406
SN <-> INT	0.567
SN <-> PBC	0.842

Table S6. Reliability and Validity Assessment of the Final Model

	Cronbach's alpha	CR	AVE
AT	0.931	0.937	0.745
BAR	0.815	0.835	0.406
BEH	1.000	1.000	1.000
CN	0.801	0.815	0.833
IDn	0.886	0.896	0.898
INT	0.922	0.924	0.719
MN	0.945	0.948	0.901
OP	1.000	1.000	1.000
VB	0.945	0.948	0.901
WW	0.938	0.939	0.891
