# Supplementary figures and images for: From Values to Action: An Integrative Explanatory Framework for Insect Conservation Intentions and Behavior
Source: Insects. 2025 Dec 15;16(12):1274. doi: 10.3390/insects16121274 (PMC12733544; doi:10.3390/insects16121274)

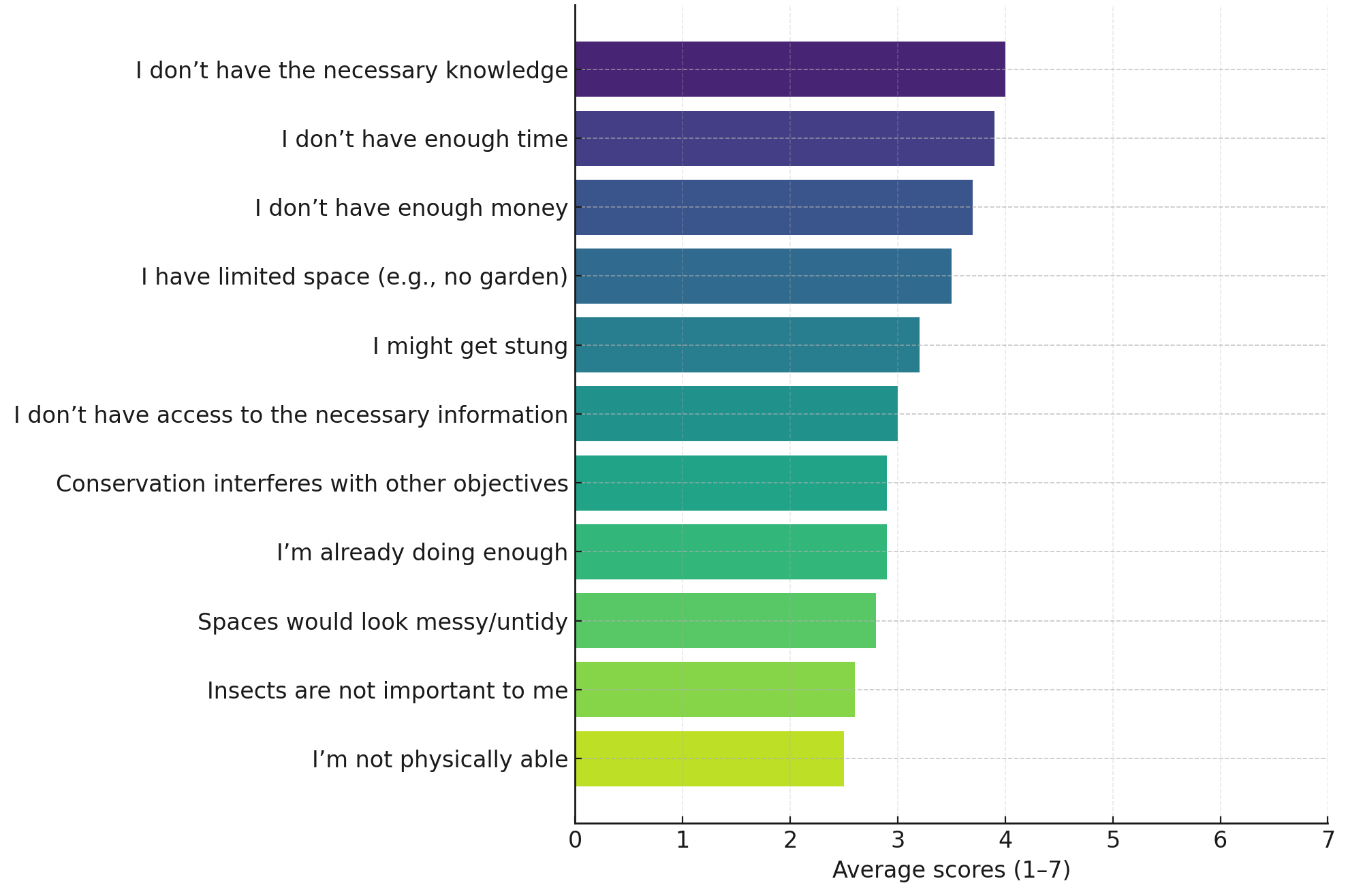

Supplement: Supplementary file 1 [file insects-16-01274-s001.zip › Figure S1. Perceived Barriers.png]
